# Sensitive and Rapid Phenotyping of Microbes With Soluble Methane Monooxygenase Using a Droplet-Based Assay

**DOI:** 10.3389/fbioe.2020.00358

**Published:** 2020-04-24

**Authors:** Hyewon Lee, Ji In Baek, Su Jin Kim, Kil Koang Kwon, Eugene Rha, Soo-Jin Yeom, Haseong Kim, Dae-Hee Lee, Dong-Myung Kim, Seung-Goo Lee

**Affiliations:** ^1^Synthetic Biology and Bioengineering Research Center, Korea Research Institute of Bioscience and Biotechnology, Daejeon, South Korea; ^2^Department of Chemical Engineering and Applied Chemistry, Chungnam National University, Daejeon, South Korea; ^3^School of Biological Sciences and Technology, Chonnam National University, Gwangju, South Korea; ^4^Department of Biosystems and Bioengineering, KRIBB School of Biotechnology, University of Science and Technology, Daejeon, South Korea

**Keywords:** synthetic biology, transcriptional factor-based biosensors, microfluidics, large-scale phenotyping, cell–cell communication

## Abstract

Methanotrophs with soluble methane monooxygenase (sMMO) show high potential for various ecological and biotechnological applications. Here, we developed a high throughput method to identify sMMO-producing microbes by integrating droplet microfluidics and a genetic circuit-based biosensor system. sMMO-producers and sensor cells were encapsulated in monodispersed droplets with benzene as the substrate and incubated for 5 h. The sensor cells were analyzed as the reporter for phenol-sensitive transcription activation of fluorescence. Various combinations of methanotrophs and biosensor cells were investigated to optimize the performance of our droplet-integrated transcriptional factor biosensor system. As a result, the conditions to ensure sMMO activity to convert the starting material, benzene, into phenol, were determined. The biosensor signals were sensitive and quantitative under optimal conditions, showing that phenol is metabolically stable within both cell species and accumulates in picoliter-sized droplets, and the biosensor cells are healthy enough to respond quantitatively to the phenol produced. These results show that our system would be useful for rapid evaluation of phenotypes of methanotrophs showing sMMO activity, while minimizing the necessity of time-consuming cultivation and enzyme preparation, which are required for conventional analysis of sMMO activity.

## Introduction

Methane-oxidizing bacteria, called methanotrophs, have been extensively investigated in biotechnological applications such as methane mitigation, environmental remediation, and biochemical production (Semrau, 2011; Shindell et al., 2012; Strong et al., 2015). Methanotrophs are the only catalysts that can convert methane in a single step under mild conditions via methane monooxygenase (MMO) (Conrado and Gonzalez, 2014). MMOs are classified into two categories: membrane-bound particulate MMO (pMMO) and cytoplasmic soluble MMO (sMMO) (Sirajuddin and Rosenzweig, 2015). While pMMO is naturally predominant and has a relatively high affinity for methane, sMMO has a higher specific activity and turnover frequency (Lawton and Rosenzweig, 2016; Ross and Rosenzweig, 2017) In addition, sMMO can use various carbon substrates including aromatics (Grosse et al., 1999). Despite its high applicability, sMMO engineering is difficult because of its unsuccessful expression in traditional laboratory strains such as *Escherichia coli* (West et al., 1992). Typically, to improve performance, methanotrophs are genetically engineered using synthetic biology approaches (Lloyd et al., 1999; Borodina et al., 2007; Crombie and Murrell, 2011; Smith and Murrell, 2011; Puri et al., 2015; Tapscott et al., 2019). In addition, a novel methanotroph strain with the desired phenotype of interest can be isolated from environmental samples.

For biocatalyst development in synthetic biology, phenotyping microbial cells with high enzyme activity often remains as the bottleneck. Although conventional methods including high-performance liquid chromatography (HPLC), gas chromatography, and mass spectrometry have been used to detect the sMMO activity (Shah et al., 1995; Grosse et al., 1999), these approaches show low sensitivity, and are time-consuming and labor-intensive (Lim et al., 2018). Phenotyping methods using naphthalene (Graham et al., 1992) or coumarin (Miller et al., 2002) shorten the detection time by facilitating parallel processing of multiple samples (∼10^3^); however, their moderate throughput renders them impractical for analyzing large-scale (∼10^6^) libraries. Therefore, highly sensitive and rapid systems are needed for large-scale quantitative phenotyping of methanotrophs with sMMO activity.

Recent advances in synthetic biology have enabled the establishment of genetically engineered biosensors for high-throughput screening of biocatalysts by converting enzyme activity into a fluorescence signal upon transcriptional activation (van der Helm et al., 2018). Few biosensors have been developed for engineering methanotroph-derived enzymes (Rohlhill et al., 2017; Selvamani et al., 2017; Lee et al., 2019). However, the target ligands were cellular metabolites such as methanol and formaldehyde, which are rapidly assimilated by methanotrophs with limited extracellular transport. Thus, applying these biosensors for phenotyping native methanotrophs is inappropriate.

This limitation can be overcome by the use of phenol-mediated genetically encoded biosensor named GESS, which can quantitatively measure sMMO activity with benzene substrate. We previously developed a GESS biosensor that uses a dimethylphenol regulator activated by phenolic compounds (Choi et al., 2014; Kim et al., 2016a, 2018; Yeom et al., 2018). GESS is not limited to detecting intracellular enzymes and has been extended to microbe phenotyping based on cell-to-cell communication (Kim et al., 2016b). In the microbe-phenotyping-GESS (MP-GESS), sensor cells surrounding the novel microbe with target enzyme activity express fluorescent reporters through extracellular transcription regulation. This versatile MP-GESS is simple and efficient; however, this culture-based assay requires long sample preparation time to obtain microbial colonies, especially for slow-growing bacteria. Also, its qualitative and labor-intensive prototyping process requires improvement.

To facilitate large-scale quantitative analysis, droplet-based microfluidics have been used for high-throughput analysis (Thorsen et al., 2001; Meyer et al., 2015; Ngara and Zhang, 2018). Microfluidic systems generate water-in-oil droplets at rates of 1000s of samples per second (Guo et al., 2012). All compartments are miniaturized bioreactors with precisely controlled reaction volumes, cell numbers, reagent concentrations, and incubation times (Joensson and Andersson Svahn, 2012). Therefore, droplet-based microfluidics has been successfully used as a screening platform with broad applications, such as for the directed evolution of enzymes, identification of novel metagenomic enzymes, and production of extracellular chemicals (Agresti et al., 2010; Hosokawa et al., 2015; Kaushik et al., 2017; Siedler et al., 2017; Giuffrida et al., 2018; de Almeida et al., 2019). It is possible to extend this technology to methanotroph phenotyping, which has not been performed before. We expect that the GESS biosensor and droplet microfluidics (GESSlet) system developed here can be used as a high-throughput screening platform for methanotroph with sMMO activity with further modifications.

Moreover, GESSlet can be extended to many applications involving cell-to-cell communication-based methanotroph phenotyping. Recently, the synthetic community of methanotrophs and non-methanotrophs has gained considerable attention as a promising platform of methane metabolism (Chistoserdova, 2018; Chistoserdova and Kalyuzhnaya, 2018). Methanotrophs can support non-methanotrophs by potentially releasing methane-derived carbons (Kalyuzhnaya et al., 2013; Tavormina et al., 2017; Yu and Chistoserdova, 2017); therefore, characterizing methanotrophs based on their communication with other microbial community members is crucial.

## Materials and Methods

### Chemicals

Chemicals and cloning reagents (restriction endonucleases, T4 DNA ligase, and DNA polymerase) were purchased from Sigma-Aldrich (St. Louis, MO, United States) and New England Biolabs (Ipswich, MA, United States), respectively. Plasmid DNA isolation and DNA extraction were conducted using plasmid preparation kits (Promega, Madison, WI, United States). Oligonucleotides were commercially synthesized and sequenced by Genotech (Daejeon, South Korea).

### GESS Preparation

As a genetic circuit system, the plasmid pGESSv4 was previously constructed with the dmpR transcriptional activator along with its own promoter, P_X_, and EGFP with the P_dmp__R_ promoter from *Pseudomonas putida* KCTC 1452 (Choi et al., 2014). To enhance the system sensitivity, the newly constructed pGESS_sfgfp_ plasmid has a constitutive P_HCE_ promoter instead of P_X_ to achieve high dmpR expression and the strong reporter gene *sf-gfp* along with the P_dmpR_ promoter. For optimization, three *E. coli* strains (DH5α, C2566, and MG1655) were used as host bacteria for pGESS_sfgfp_. For phenotyping of wild-type methanotrophs with high reliability, the GESS_sfgfp_ genetic construct was integrated into the *E. coli* DH5α chromosome as described previously (Kim et al., 2018; Yeom et al., 2018). The *E. coli* sensor cells were cultured in Luria-Bertani medium at 37°C and stored at −80°C in 15% glycerol until further analysis.

To use the GESS microbial sensor, the frozen cell stock was thawed on ice and plated to grow single colonies, which were inoculated in 5 mL of Luria-Bertani medium at 37°C for 16 h. After culturing the cells, the optical density of the bacterial suspension was measured at 600 nm with a spectrophotometer. The cells were harvested by centrifugation at 1000 × *g* for 20 min and then resuspended in M9 medium (47.8 mM Na_2_HPO_4_, 22 mM KH_2_PO_4_, 18.7 mM NH_4_Cl, 8.6 mM NaCl, 2 mM MgSO_4_, 0.1 mM CaCl_2_, 0.1% acetate, and 0.01% thiamine) with 10–20 mM benzene.

### Methanotroph Culture

Five methanotrophs strains were obtained: *Methylococcus capsulatus* Bath from American Type Culture Collection (ATCC 33009, Manassas, VA, United States), *Methylosinus sporium* 5 and *Methylocella silvestris* BL2 from Deutsche Sammlung von Mikroorganismen und Zellkulturen (DSMZ 17706 and 15510, Braunschweig, Germany), *Methylosinus trichosporium* OB3b from Korean Collection for Type Cultures (KCTC 12760, Jeollabuk-do, South Korea), and *Methylomonas* sp. DH-1 from Eun Yeol Lee (Kyung Hee University, Yongin-si, South Korea). All methanotrophs were cultured in nitrate mineral salts (NMSs) medium, pH 6.8 or pH 5.8 (*M. silvestris* BL2), as described previously (Whittenbury et al., 1970). Frozen stocks of methanotroph in 10% (v/v) dimethyl sulfoxide were thawed, washed, and inoculated into 3 mL NMS media in glass bottles. The bottles were sealed with rubber stoppers and aluminum caps. The headspace was filled with a methane-air gas mixture (30:70), and the cultures were incubated at 30 or 42°C with agitation (200 rpm). After 24 h, the methanotroph culture was transferred to 200-mL sealed serum bottles containing 30 mL of NMS medium. Under a methane-air gas mixture, the cultures were incubated at 30 or 42°C with agitation for 2 days. Cells were harvested by centrifugation at 1000 × *g* for 10 min and resuspended at the desired concentration in M9 medium.

### Operation of the Microfluidic Device

For high-throughput co-encapsulation of methanotrophs and GESS, we used the μEncapsulator 1–2 Reagent Droplet Chip (50 μm, fluorophilic, Dolomite Microfluidics, Royston, United Kingdom). Monodisperse droplets with diameters of 40–50 μm were produced at the flow focusing junction, which has two aqueous inlets, two oil inlets, and an outlet channel. This chip interface interacts directly with the Sample Reservoir Chip (Dolomite Microfluidics), where the methanotroph sample and GESS/benzene solution were loaded through separate channels. Both channels were connected to a single pressure pump through PTFE tubing (Kinesis, Berlin Township, NJ, United States) with a Y-connector. The tubing sections were filled with FC-40 oil (Sigma-Aldrich), which served as the displacement fluid. By precisely controlling the pressure pump, a pressure of 300 mbar was used for aqueous sample delivery. An oil phase consisting of FC-40 with 2% Pico-Surf (Dolomite Microfluidics) was delivered through a single port of the microfluidic chip using a pressure pump at 500 mbar, and the flow was split equally into the two channels. Pico-Surf, a fluorinated surfactant, was added to stabilize the droplets.

### Fluorescence Plate Reader Analysis

To validate the GESS biosensors, GESS cells reacted with or without *M. trichosporium* cells in M9 media containing 1–20 mM benzene at 37°C were analyzed with the Victor X multilabel plate reader (PerkinElmer, Waltham, MA, United States) or Infinite 200 PRO multi-functional microplate reader (Tecan, Männedorf, Switzerland) using black-walled 96-well polystyrene plates at excitation and emission wavelengths of 485 and 535 nm, respectively. To determine the specific fluorescence, the optical density was determined by measuring the absorbance at 600 nm in a Victor X multilabel plate reader or Infinite 200 PRO multi-functional microplate reader.

### HPLC Analysis

To verify sMMO activity for the conversion of benzene to phenol, *M. trichosporium* cells were treated with benzene in NMS medium, and phenol formed in the reaction mixture was analyzed by HPLC (1260 infinity, Agilent Technologies, Santa Clara, CA, United States) fitted with an Agilent Zorbax Eclipse XDB-C18 column. The mobile phase comprising a mixture of acetonitrile and water (50:50) was delivered at a flow rate of 1 mL/min at 23°C. The UV absorbance of the samples was measured at 270 nm.

### Microscopic Observation

Droplets containing GESS and methanotrophs were incubated at 37°C for 5 h. Droplets were imaged using an Axiovert A1 inverted microscope (Carl Zeiss, Oberkochen, Germany) equipped with a GFP filter (excitation at 455–495 nm, emission at 505–555 nm). Images were analyzed using ImageJ software (National Institutes of Health, Bethesda, MD, United States) (Schneider et al., 2012). Further, methanotrophic bacteria in droplets were imaged and analyzed at 20 or 63x magnification in bright-field mode with the Axiovert A1 inverted microscope.

### Flow Cytometry Analysis

After incubation at 37°C for 5 h, the droplets were broken by adding 200 μL of 1H, 1H, 2H, 2H-perfluoro-1-octanol. The sample tube was agitated and centrifuged for 30 s at 1000 × *g*. The supernatant was transferred to 1 mL of phosphate-buffered saline. Fluorescence intensity was measured with a FACSAria^TM^ Flow Cytometer Sorter (BD Biosciences, Franklin Lakes, NJ, United States) with a blue laser source (488 nm). Data were acquired using BD CellQuest Pro (version 4.0.2, BD Biosciences) and analyzed using FlowJo software (Tree Star, Ashland, OR, United States). Debris were excluded via forward and side scattering measurements. The target intensity was calculated by averaging the total fluorescence of GESS cells that had been reacted with methanotrophs and benzene. The fluorescence of GESS cells without methanotroph was averaged to calculate the background signal. The net signal was determined by subtracting the background signal from the target signal.

## Results and Discussion

### Assay Development

**FIGURE 1:**
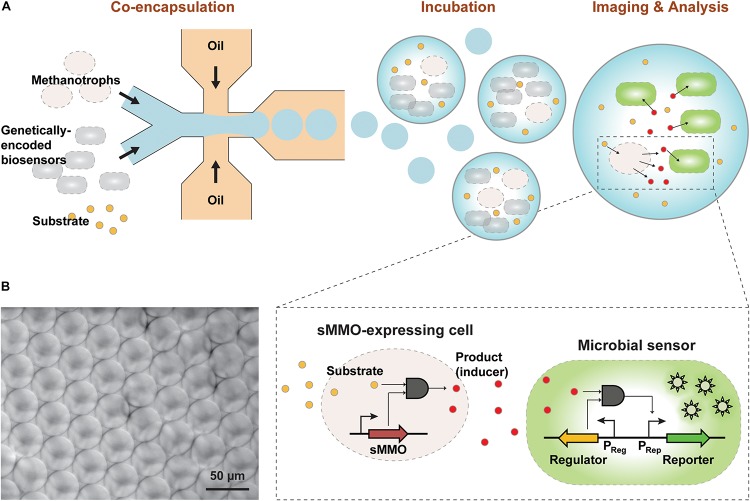
**(A)** Overview of the GESSlet assay workflow. In the first assay step, methanotrophs and genetically encoded microbial sensors were co-encapsulated into monodisperse water-in-oil droplets with the substrate using a microfluidic system. During incubation at 37°C, methanotrophs with sMMO activity converted benzene to phenol, which activated the transcription factor and expression of the fluorescent reporter in the GESS biosensor. Thereafter, the fluorescence signal was measured with a microscope and by flow cytometry. **(B)** Optical micrograph of the droplets produced with minimum variation.

**FIGURE 2:**
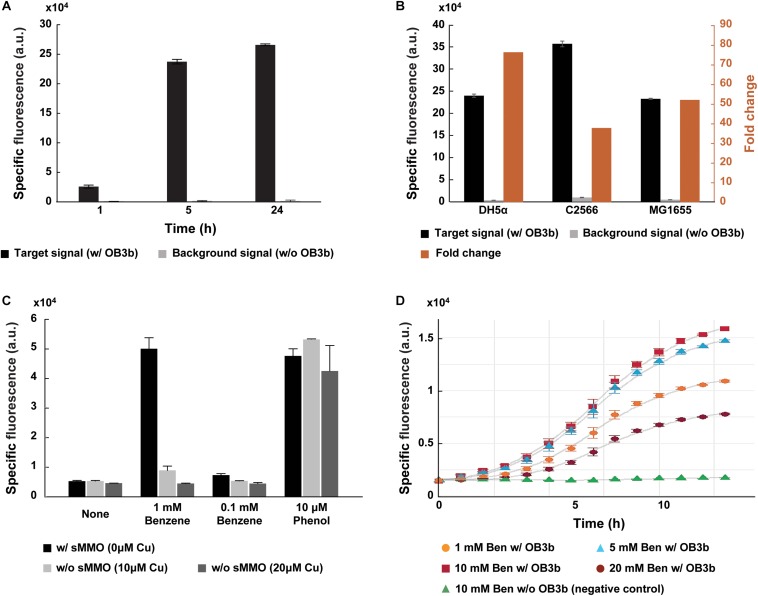
Specific detection of sMMO in methanotroph *Methylosinus trichosporium* OB3b via GESS biosensors. **(A)** Comparison of the specific fluorescent signal with and without *M. trichosporium* cells. In the presence of *M. trichosporium* cells, fluorescence intensity increased with time. **(B)** Optimization of the host for the GESS biosensor. The DH5α strain revealed the highest fold-change. **(C)** Bar graphs representing incubation with and without copper, which differentiates the expression and activity of sMMO and pMMO in methanotrophs. Without copper, sMMO expressed in methanotrophs specifically induced the fluorescent signal of GESS with the benzene substrate. Phenol was used as a positive control, generating fluorescent signals in all cases regardless of copper concentration. **(D)** Optimization of benzene concentration. In all cases, the OD values were 1.0 (GESS biosensors) and 0.1 (*M. trichosporium* OB3b), respectively. Error bars represent standard deviation (*n* = 3–5).

To quantify sMMO activity in methanotrophs based on cell-to-cell communication, we applied our transcription factor-based microbial sensor, GESS. A previous study reported that the GESS sensor enabled identification of phenol-generating enzymes in a high-throughput manner. sMMO converts the substrate benzene into phenol, which diffuses into GESS sensor cells to induce the expression of a fluorescent reporter through extracellular transcription regulation (Figure 1). In an initial trial with *M. trichosporium* OB3b, the fluorescence signal intensity increased over time (Figure 2A). Also, liquid chromatography analysis indicated that phenol produced by *M. trichosporium* increased over time with either 1 or 10 mM benzene as the substrate. Moreover, *M. trichosporium* did not degrade phenol for 24 h (see Supplementary Figure 1). This indicates that phenol was generated by sMMO-catalyzed oxidation of benzene and not degraded by the methanotroph or GESS metabolism, unlike methanol and formaldehyde. Indeed, *in vivo* MMO activity is typically measured by oxidizing substrates other than methane, such as propylene, to prevent further metabolism of the product. Thus, by using phenol-mediated GESS, the *in vivo* activity of sMMO in microbes can be measured. Notably, negative control strains such as *E. coli* DH5 and *Mesorhizobium loti* showed negligible signals with 1 mM benzene (Supplementary Figure 2) due to an absence of monooxygenase activity.

In this assay, for sensitive measurement of sMMO, we engineered the GESS plasmid by replacing the reporter gene with *sf-gfp* and promoter for a transcription factor under a strong constitutive promoter P_HCE_. Furthermore, we empirically selected the host cell for the GESS biosensor for robust detection. The host cell greatly influences the assay duration, sensitivity, and specificity as a device containing genetic circuit-based sensing molecules (Wang et al., 2013). Both the *E. coli* B and K strains have been widely used to develop genetically encoded biosensors and produce recombinant proteins. As shown in Figure 2B, GESS with the B strain such as C2566 showed a lower fold-change (38-fold) because of its high background signal compared to K-12 strains such as DH5α and MG1655. The DH5α strain showed the highest fold-change (76-fold). Additionally, to reliably detect sMMO in methanotrophs, we integrated the GESS genetic construct into the DH5α chromosome to minimize cellular heterogeneity due to variations in the copy numbers of the sensing circuit gene.

Phenol-mediated GESS enables specific detection of sMMO in methanotrophs. Of the two MMOs in methanotrophs, sMMO can oxidize not only methane but also various substrates including aromatic compounds. To evaluate the specificity of GESS, we tuned sMMO and pMMO expression using copper at different concentrations, which reciprocally regulates the expression of sMMO and pMMO in methanotrophs (Murrell et al., 2000). As shown in Supplementary Figure 3a, SDS-PAGE shows protein bands corresponding to sMMO and pMMO. *M. trichosporium* cells produced sMMO in the absence of copper. When the copper concentration was high (10 and 20 μM of copper), copper enhanced pMMO expression and formation of the intracytoplasmic membrane in *M. trichosporium* as observed in previous studies (Collins et al., 1991; Nielsen et al., 1996; Semrau et al., 2010; Dassama et al., 2017) (Supplementary Figure 3b). When *M. trichosporium* cells were cultured at various copper concentrations, we introduced GESS sensors to the reaction tube and analyzed the fluorescence signal intensity after incubation. Notably, positive (10 μM phenol) and negative (none – no substrate) control signals from different copper concentrations exhibited negligible differences. As shown in Figure 2C, our assay scheme yielded a measurable signal only in the absence of copper when 1 mM benzene was used as the substrate. However, *Methylocella* strains are known to constitutively express sMMO even when cultured with copper. The GESS biosensors were thus applied to measure sMMO activity in *Methylocella silvestris* BL2 cultured with and without 10 μM copper. With 1 mM benzene, both showed significant signals (Supplementary Figure 4), demonstrating the high specificity of GESS for detecting methanotrophs with sMMO activity.

Moreover, we predicted that 1% of benzene was converted to phenol because the fluorescence intensities were similar on using 1 mM benzene without copper and 10 μM phenol (Figure 2C). In this context, 0.1 mM benzene without copper may be converted to 1 μM phenol, resulting in a minimally distinguishable signal. We further optimized the benzene concentration to maximize GESS biosensor sensitivity for sMMO detection. The signals from the negative controls (no *M. trichosporium* cells) with 1–20 mM benzene displayed negligible differences in intensity (Supplementary Figure 5). For *M. trichosporium* cells cultured without copper, the fluorescent signal increased when the benzene concentration was increased from 1 to 10 mM; however, the signal decreased drastically when 20 mM benzene was used, possibly due to the chemical toxicity (Figure 2D). Thus, we used 10 mM benzene for all subsequent analyses.

### Integration of GESS-Based sMMO Detection With Droplet Fluidics

Using the GESS-based assay for analyzing methanotrophs with sMMO presents several practical challenges. Using agar plates, a previous study reported that GESS biosensors enabled easy and simple identification of active clones with desired enzyme activity (Kim et al., 2016b); however, colony-based detection methods showed relatively poor dynamic ranges (Leemhuis et al., 2009). In addition, this method relies on a time-consuming cell culture step that requires days to weeks for phenotyping of slow-growing bacteria. To improve the assay quality and throughput of GESS-based sMMO detection, we used a droplet-based microfluidic technique for isolating methanotrophic cells in picoliter-sized compartments with GESS biosensors (Figure 1A). In the present GESSlet (GESS biosensor and droplet microfluidics), two different aqueous and oil phases were delivered into a glass microfluidic device (Dolomite) using pressure pumps. A few 1000 45-μm water-in-oil droplets were produced in 1 s with minimum variations (coefficient of variation of 0.59–3.22%, Figure 1B). Enzymatic turnover of benzene in a confined compartment increased the phenol product concentrations, thus improving the assay sensitivity. Additionally, we optimized the OD of GESS biosensors, which determines the number of sensor cells encapsulated. As the GESS OD decreased, the phenol-induced target signal was decreased significantly whereas the change in the background signal was negligible. Thus, as shown in Supplementary Figure 6, the optimal OD of GESS biosensors is 1–2. We determined that OD ∼1.0 (∼50 cells encapsulated per droplet) maximizes fold change and minimizes droplet-to-droplet variability for reliable measurement. Moreover, this assay platform requires only small sample volumes (<100 μL) for 1000s of measurements, which is beneficial for methanotroph phenotyping by overcoming the challenges in culturing.

**FIGURE 3:**
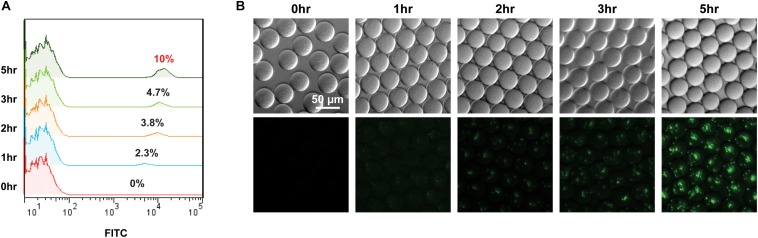
Time course signal increase of GESS sensor integrated into the droplet-based microfluidic (GESSlet) system. **(A)** Flow cytometric analysis revealing an increased fluorescence intensity of GESS to the right as a function of reaction time. Nearly all GESS biosensors (∼10% of total cells) yielded measurable signals after 5 h. **(B)** Representative images of droplets encapsulated with GESS and *M. trichosporium* cells. Fluorescence intensities in droplets increased over time.

Thereafter, we evaluated the compatibility of GESS with the droplet system. GESS biosensors and *M. trichosporium* cells mixed at a 1:10 ratio were isolated in droplets with 10 mM benzene. After incubation for 1–5 h at 37°C, co-cultured cells (10% GESS and 90% *M. trichosporium*) were recovered from the droplets and their fluorescence values were measured by flow cytometry. As illustrated in Figure 3A, the proportion of fluorescently labeled cells gradually increased with incubation time. Particularly, we observed that nearly 10% of all cells showed fluorescence after 5 h, indicating that all GESS cells responded. Moreover, time-lapse imaging of GESS biosensors in the droplet revealed that fluorescence indeed increased within every droplet as the incubation time was increased (Figure 3B). The reaction time was set to 5 h to maximize sensitivity, while minimizing the assay duration. If necessary, this assay duration can be further reduced and still yield reasonable signals after 1 h of incubation. Based on these results, the droplet system was successfully integrated with the GESS biosensor for efficient measurement of sMMO with minimal sample preparation.

### Detection Performance

For phenotyping analysis of microbes with desired enzyme activity, sensitivity is most relevant in the context of the total number of cells required for quantification. To investigate the sensitivity of the GESSlet system, we first calibrated the number of cells per droplet. Cell encapsulation in droplets is a random process that relies on the cell density in the suspension. We calculated the cell number in each droplet from the cell density measured in bulk solution and droplet volume measured using microscopic imaging analysis. A previous study predicted that a 40-pL droplet contains four methanotrophic cells on average, as 0.1 OD of inoculum corresponds to ∼9.28 × 10^7^ cells/mL (Myung et al., 2016). We experimentally verified the number of cells encapsulated in a single droplet through microscopic observation. GESS cells showing fluorescence following a pre-reaction with phenol were encapsulated at different concentrations. At each concentration (input OD = 0.02, 0.05, and 0.1), the average number of cells in each droplet was 0.8, 1.8, and 4.1 cells/droplet, respectively (Supplementary Figure 7), which agrees with the theoretical calculation (0.9, 2.2, and 4.4 cells/droplet). In addition, a Poisson distribution of cell numbers was observed to be consistent with the random distribution of cells within the droplets.

**FIGURE 4:**
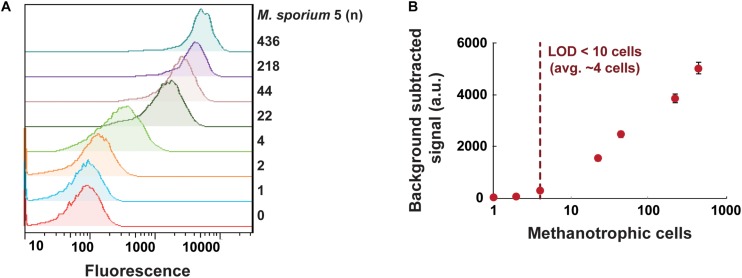
Sensitive quantification of sMMO in methanotrophs. **(A)** Fluorescence intensity profiles of GESS microbial sensors showing the methanotroph (*Methylosinus sporium* 5) dose-dependent response. **(B)** Calibration curve of *M. sporium* 5 cells using the droplet-based GESS sensor. The limit of detection was projected to be an average of 4 cells in a droplet. Error bars represent the standard error of the mean.

We next examined the sensitivity of the GESSlet system by plotting a calibration curve. In each droplet containing GESS sensors with 10 mM benzene, we incubated an average of 0–436 methanotrophic cells for 5 h at 37°C. After chemically breaking the droplets, the fluorescent signal of individual cells was quantified by flow cytometry. As shown in Figure 4A, GESS revealed a methanotroph-dose-dependent response; the fluorescence signal increased with an increasing number of methanotroph cells. From the calibration curve shown in Figure 4B, the assay revealed that the methanotroph detection limit was 4 cells per droplet on average for identifying sMMO activity. Considering the variation in encapsulated cells, sMMO activity can be measured from at least 10 methanotrophic cells, which is substantially lower than that using a conventional assay platform with a microplate reader (Supplementary Figure 8). This indicates that the GESS integrated with droplet fluidics is highly sensitive.

This sensitivity can be further enhanced using a modified GESS with higher sensitivity (Choi et al., 2014) or incorporating the amplification of target methanotrophs in a droplet. Water-in-oil emulsions provided favorable conditions for methanotroph culturing with enhanced methane delivery without agitation, thus successfully achieving methanotroph growth (Myung et al., 2016). Moreover, our GESSlet system requires only a few rounds of generations derived from a single cell. Thus, we expect that our versatile system may be extended to single-cell analysis and high-throughput screening for methanotrophs with sMMO activity with substantial further development.

### Functional Phenotyping of Methanotrophs With sMMO Activity

**FIGURE 5:**
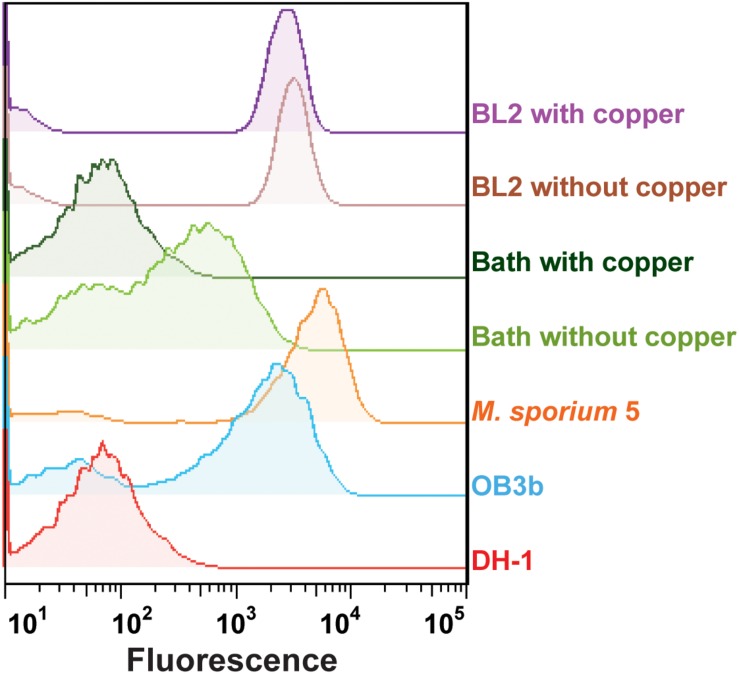
Quantification of sMMO activity in five different methanotrophs. *Methylomonas* sp. DH-1 cells expressing only pMMO showed a minimal fluorescent signal, whereas the other four methanotrophs with sMMO showed relatively higher intensities. In the presence of copper, the GESS signals of *Methylococcus capsulatus* (Bath) with pMMO were decreased to baseline levels, whereas measurable signals were observed with *Methylocella silvestris* BL2 from the GESS biosensors.

To verify the applicability of the GESSlet, we performed functional phenotyping for five different methanotrophic bacteria based on their sMMO activities. Each methanotroph strain loaded into the microfluidic chip was successfully encapsulated in a water-in-oil droplet with GESS sensors and 10 mM benzene. Following off-chip incubation, the droplets were broken into a continuous aqueous phase, and the fluorescence intensity was measured. As expected, the GESS biosensors showed prominent signals from the three methanotrophs (*Methylococcus capsulatus* (Bath) and *M. trichosporium*, *Methylosinus sporium* 5, and *M. silvestris* BL2) with different sMMO activities, whereas GESS with *Methylomonas* sp. DH-1 displayed minimum background fluorescence (Figure 5). In addition, *M. capsulatus* cells cultured with 10 μM copper generated a negligible fluorescence signal from the GESS sensors because of the absence of sMMO activity. On the contrary, *M. silvestris* BL2 yielded a fluorescence signal both when cultured with and without copper because of its copper-independent expression of sMMO. Moreover, sMMO of *M. trichosporium* cells showed higher specific activity than that of *M. capsulatus*, which agrees with a previous report (Shaofeng et al., 2007). Therefore, the GESSlet successfully quantified different levels of sMMO activity from various methanotrophs, demonstrating its potential applications for methanotroph screening.

To apply this system in methanotroph screening in the future, the procedure of phenotyping and sorting of microbes of interest needs to be further improved. A gelling agent could be incorporated into our flexible assay system for imaging and sorting of GESS-methanotroph compartments by flow cytometry in a high-throughput manner (Duarte et al., 2017). Addition of gelling reagents would make the GESSlet a powerful system for screening desired methanotrophs for developing industrially applicable enzymes and engineering microbial cell factories based on cell-to-cell communication. During an initial trial of screening natural samples, we isolated *Hydrogenophaga* strains, which are reported to have a symbiotic relationship with methanotrophs. Previous studies have reported that *Hydrogenophaga* strains are co-cultured during a methanotroph isolation process. This indicates the possible applicability of our GESSlet system.

In summary, we developed a technique for cell-to-cell communication-based phenotyping of methanotrophs with sMMO activity using a genetically designed a whole-cell biosensor named GESS and a droplet microfluidic system. sMMO has attracted attention from applied microbiologists and biochemical engineers because of its broad substrate specificity. Our GESS biosensor has many advantages for identifying methanotrophs with high sMMO activity, such as its ability to observe *in vivo* sMMO activity without metabolic inhibition and its high specificity. Additionally, this assay demonstrates high throughput and highly sensitive measurement, which is beneficial in quantitative large-scale analysis. For application to the screening of methanotrophs with sMMO activity, it is possible that gelling agents could be incorporated into the GESSlet system for efficiently and rapidly sorting compartments of GESS-methanotrophs of interest. This would be beneficial for the screening of large-scale libraries of methanotrophs, which would typically be very time-consuming (weeks or possibly months) using conventional methods. Moreover, the choice of partner microbe in our system is highly flexible. Thus, this versatile system can be easily extended to many other studies such as the characterization of the co-metabolism of methanotroph–non-methanotroph communities.

## Data Availability Statement

The datasets generated for this study are available on request to the corresponding author.

## Author Contributions

HL designed and performed the experiments, analyzed the data, and drafted the manuscript. JB, SK, and KK performed the experiments. ER, S-JY, HK, D-HL, and D-MK conceived the research and assisted in data interpretation. S-GL conceived the initial project, assisted in research design, and analyzed the data, and revised the manuscript. All authors reviewed the manuscript.

## Conflict of Interest

The authors declare that the research was conducted in the absence of any commercial or financial relationships that could be construed as a potential conflict of interest.
