# Supplementary material for: Sensitive and Rapid Phenotyping of Microbes With Soluble Methane Monooxygenase Using a Droplet-Based Assay
Source: Front Bioeng Biotechnol. 2020 Apr 24;8:358. doi: 10.3389/fbioe.2020.00358 (PMC7193049; doi:10.3389/fbioe.2020.00358)
Supplement: Supplementary file 1 [file Data_Sheet_1.PDF]

Supplementary Material

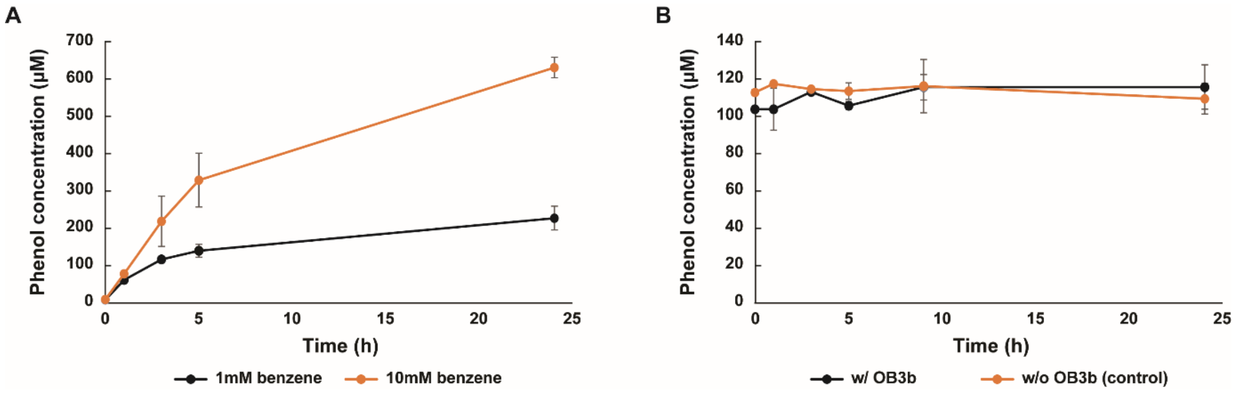

Supplementary Figure 1. (**a**) Conversion of benzene to phenol by *Methylosinus trichosporium* OB3b. Phenol concentration increased with time. (**b**) Phenol reactivity in *M. trichosporium* OB3b-expressing cells. There was no significant change in the phenol concentration over time, indicating that *M. trichosporium* OB3b did not degrade phenol. In all cases, the absorbance values of *M. trichosporium* OB3b cells were 0.9. Error bars represent standard deviation (n = 3).

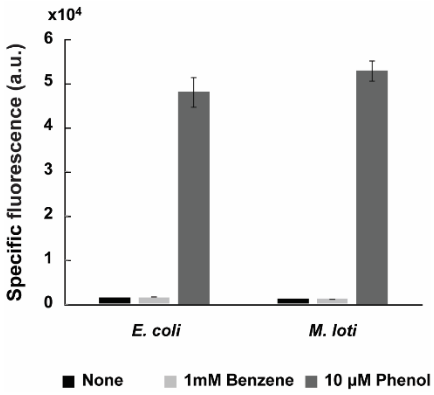

Supplementary Figure 2. Negative control strain measurements using GESS sensors. Both *Escherichia coli* (Gammaproteobacteria) and *Mesorhizobium loti* (Alphaproteobacteria) showed negligible signals from GESS sensors with 1mM benzene substrate. Notably, 10 μM of phenol (positive control) yielded measurable signals. Error bars represent the standard deviation (n = 3).

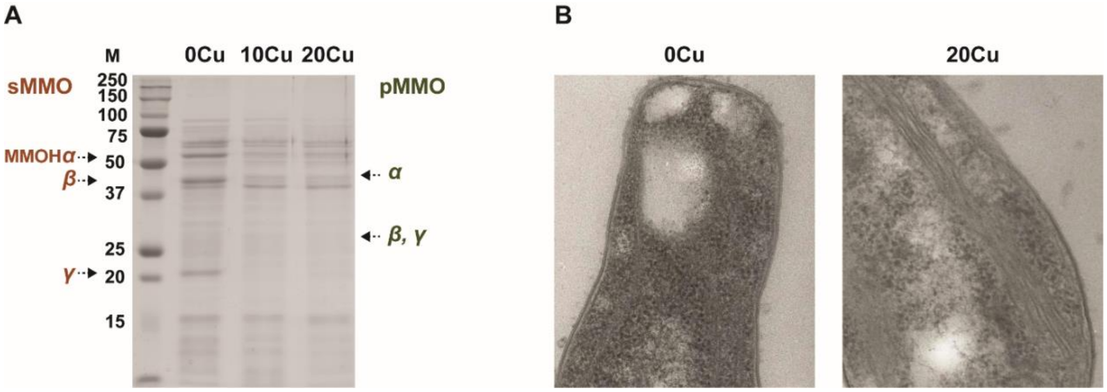

Supplementary Figure 3. Effects of copper on soluble methane monooxygenase (sMMO) expression in methanotrophs. (**a**) SDS-PAGE analysis of *M. trichosporium* OB3b in cells cultured with copper at 0 µM (0Cu), 10 µM (10Cu), and 20 µM (20Cu). sMMO was expressed in the absence of copper, whereas particulate MMO was produced in the presence of copper. (**b**) Transmission electron microscopy images of *M. trichosporium* OB3b cells in the absence and presence of copper, showing an increase in the inner membrane content at high copper concentrations.

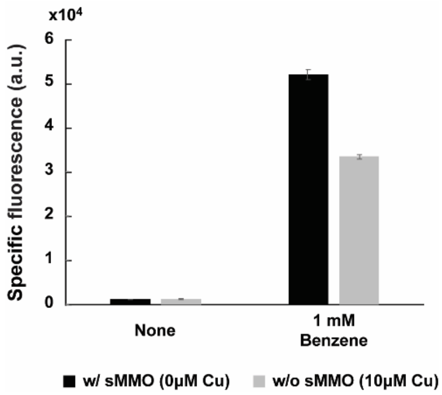

Supplementary Figure 4. Measurement of sMMO activity in *Methylocella silvestris* BL2 using the GESS biosensor. sMMO is constitutively expressed in *M. silvestris* BL2 regardless of copper concentration. With 1 mM benzene as the substrate, the GESS biosensor produced measurable signals from *M. silvestris* BL2 cultured from both with and without copper. Error bars represent the standard deviation (n = 3).

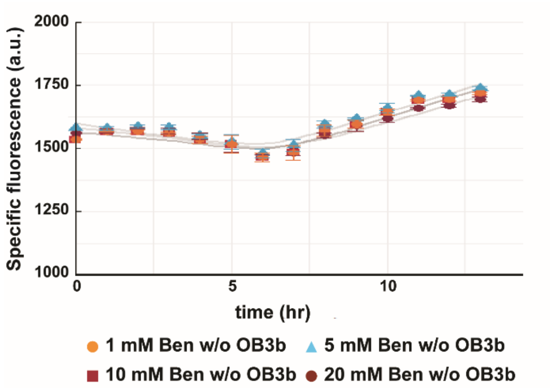

Supplementary Figure 5. Comparison of specific fluorescence signals from negative controls with 1–20 mM benzene. In the absence of *M. trichosporium* OB3b, the specific fluorescence differed negligibly at varying benzene concentrations. Error bars represent standard deviation (n = 3).

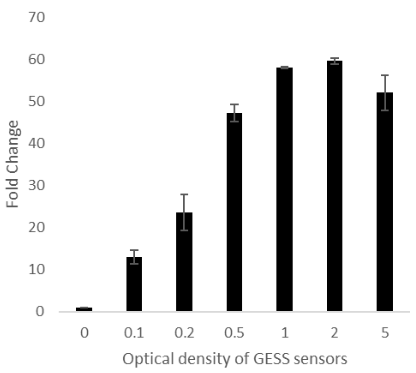

Supplementary Figure 6. Detection of fold changes in accordance with the biosensor OD. The optimal is 1-2 OD of GESS biosensors to maximize fold change. As the OD of GESS sensors was decreased from 1 or increased from 2, fold change was decreased due to the lower target signal rather than background signal. Error bars represent the standard deviation (n = 3).

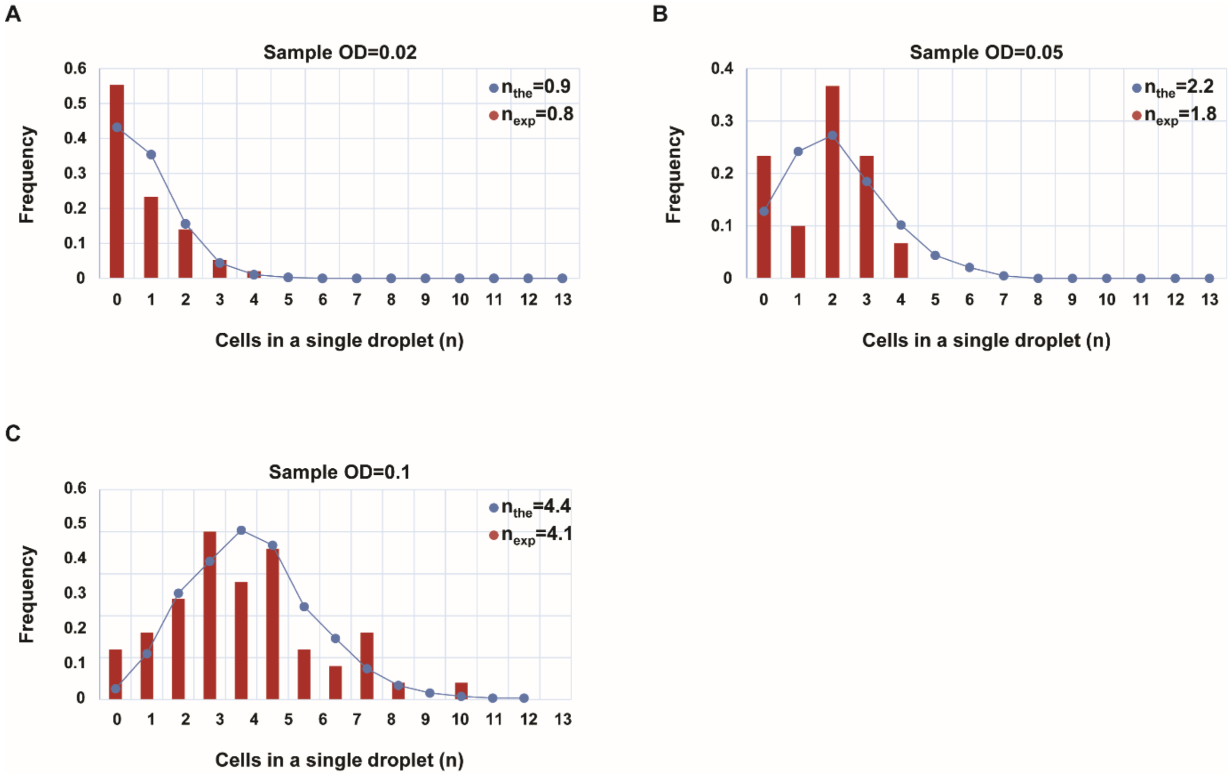

Supplementary Figure 7. Theoretical and experimental characterization of droplet occupancy with cells. Bar graphs show the frequency of cells encapsulated in a single droplet from samples with absorbance values of (**a**) 0.02, (**b**) 0.05, and (**c**) 0.1. Each probability histogram fits well with a Poisson distribution.

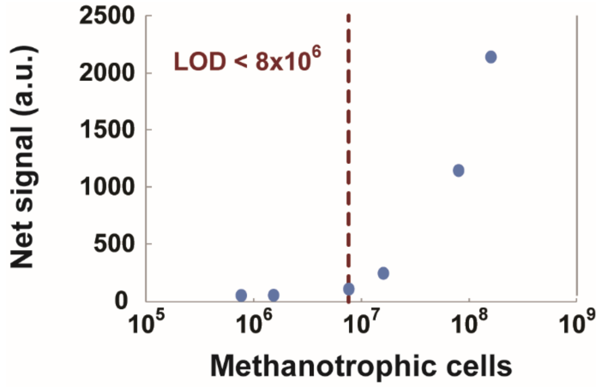

Supplementary Figure 8. Calibration curve of *Methylosinus sporium* 5 using a conventional multi-well plate. The limit of detection was projected to be approximately 8×10^6 cells in a well.
